# Body Composition Changes During a 24-h Winter Mountain Running Race Under Extremely Cold Conditions

**DOI:** 10.3389/fphys.2019.00585

**Published:** 2019-05-14

**Authors:** Daniela Chlíbková, Alena Žákovská, Thomas Rosemann, Beat Knechtle, Josef Bednář

**Affiliations:** ^1^Centre of Sports Activities, Brno University of Technology, Brno, Czechia; ^2^Institute of Experimental Biology, Masaryk University, Brno, Czechia; ^3^Institute of Primary Care, University of Zurich, Zurich, Switzerland; ^4^Medbase St. Gallen Am Vadianplatz, St. Gallen, Switzerland; ^5^Institute of Mathematics, Brno University of Technology, Brno, Czechia

**Keywords:** extreme weather conditions, body composition, winter, ultra-running, 24 h

## Abstract

**Background:** To date, no study has focused on body composition characteristics and on parameters associated with skeletal muscle damage and renal function in runners participating in a 24-h winter race held under extremely cold environmental conditions (average temperature of -14.3°C).

**Methods:** Anthropometric characteristics, plasma urea (PU), plasma creatinine (Pcr), creatine kinase (CK), plasma volume (PV) and total body water (TBW) were assessed pre- and post-race in 20 finishers (14 men and 6 women).

**Results:** In male runners, body mass (BM) (*p* = 0.003) and body fat (BF) (*p* = 0.001) decreased [-1.1 kg (-1.4%) and -1.1 kg (-13.4%), respectively]; skeletal muscle mass (SM) and TBW remained stable (*p* > 0.05). In female runners, BF decreased (*p* = 0.036) [-1.3 kg (-7.8%)] while BM, SM and TBW remained stable (*p* > 0.05). The change (Δ) in BM was not related to Δ BF; however, Δ BM was related to Δ SM [*r* = 0.58, *p* = 0.007] and Δ TBW (*r* = 0.59, *p* = 0.007). Δ SM correlated with Δ TBW (*r* = 0.51, *p* = 0.021). Moreover, Δ BF was negatively associated with Δ SM (*r* = -0.65, *p* = 0.002). PV (*p* < 0.001), CK (*p* < 0.001), Pcr (*p* = 0.004) and PU (*p* < 0.001) increased and creatinine clearance (CrCl) decreased (*p* = 0.002). The decrease in BM was negatively related to the increase in CK (*r* = -0.71, *p* < 0.001). Δ Pcr was positively related to Δ PU (*r* = 0.64, *p* = 0.002). The decrease in CrCl was negatively associated with the increase in both PU (*r* = -0.72, *p* < 0.001) and CK (*r* = -0.48, *p* = 0.032).

**Conclusion:** The 24-h running race under extremely cold conditions led to a significant BF decrease, whereas SM and TBW remained stable in both males and females. Nevertheless, the increase in CK, Pcr and PU was related to the damage of SM with transient impaired renal function.

## Introduction

To date, there is a limited number of studies on ultra-endurance athletes competing in cold temperatures ranging from -21°C to -2°C (Stuempfle et al., 2002, 2003; Chlíbková et al., 2019) and under very cold weather conditions with temperatures down to -48°C (O’Hara et al., 1977; Paulin et al., 2015; Coker et al., 2017; Johannsen et al., 2018; Schalt et al., 2018).

Factors such as anthropometric characteristics [e.g., body fat (BF)] of the athlete make a difference to the strategies that can be adopted to offset any negative impact of cold environment on athletic performance (Nimmo, 2004). Individuals with lower BF are prone to greater heat loss and higher energy expenditure when exposed to cold (Halsey and Stroud, 2012). Since respiratory water loss is greater in the cold and diuresis is a possible route of fluid loss (Nimmo, 2004), a considerable reduction in body mass (BM) during exercise in the cold can be expected. A case study of competitors undertaking an antarctic ultra-endurance event showed a reduction in both BM and lean mass (Paulin et al., 2015). On the contrary, recent studies in the cold antarctic environment (Coker et al., 2017; Johannsen et al., 2018; Schalt et al., 2018) demonstrated preserved fat free mass in the athletes.

In ultra-marathon running, 24-h ultra-marathons enjoy great popularity (Fellmann et al., 1988; Kao et al., 2008; Knechtle et al., 2010b, 2011b; Waśkiewicz et al., 2012; Chlíbková et al., 2014, 2019; Costa et al., 2014). In the existing literature, the ambient temperature seems to be of importance for the changes in BM during such a race. Generally, BM decreases during a 24-h ultra-marathon. It has been shown that 26% of the runners lost more than 7% of their baseline BM during a 24-h ultra-marathon held at moderate temperatures (12–15°C) (Kao et al., 2008). BM losses during a 24-h ultra-marathon were observed by Fellmann et al. (1988), Knechtle et al. (2010b), Chlíbková et al. (2014) and Costa et al. (2014) in their studies on races held at temperatures ranging from 0 to 20°C. The decrease in BM correlated with the decrease in BF in a study of 24-h ultra-marathoners competing at temperatures of 10–31°C (Knechtle et al., 2011b). Recent studies demonstrated that top finishers of ultra-endurance races often finish with BM losses over 3–4% (Hoffman and Stuempfle, 2014; Hoffman et al., 2018) and 1% of BM loss could be from fat utilization (Stuempfle et al., 2011).

On the other hand, skeletal muscle volume expansion, total body water (TBW) increase and the occurrence of peripheral edema have been reported after ultra-running events (Bracher et al., 2012; Cejka et al., 2012) and it has been suggested that the increase in TBW might be involved in the fluid shift to distal compartments. Vitiello et al. (2015) noted that the increases in circumferences and in hydric volume were associated with contractile impairment in the calf during an extreme mountain ultra-marathon. An ultra-endurance run can lead to substantial skeletal muscle damage and acute inflammatory response as evidenced by a wide range of muscle injuries (Kratz et al., 2002). Fellmann et al. (1988) demonstrated that a 24-h running race caused more muscular lesions than a triathlon with a considerable increase in creatine kinase (CK). Plasma urea (PU) significantly increased after a 1600-km ultra-marathon in the study by Fallon et al. (1999) and the authors suggested a catabolic state indicating skeletal muscle damage. Skenderi et al. (2006) stated that even a moderate intensity during a prolonged exercise can induce exertional rhabdomyolysis.

To the best of our knowledge, no studies have investigated the changes in body composition in athletes competing for 24 h in extremely cold winter conditions with an average temperature of -14.3°C. There is no available data on changes in body composition and parameters associated with the damaging process leading to cellular injuries in athletes running for 24 h in extremely cold winter conditions. With this in mind, the aim of the study was to quantify changes in body composition related to skeletal muscle mass (SM) damage using diffferent methods. We hypothesized that a 24-h winter mountain running race would lead to decreases in both BM and BF and might be the cause of body fluid shifts to extracellular compartments.

## Materials and Methods

### Ethics Statement

The study received ethical approval from the institutional review boards of the Centre of Sport Activities at the Brno University of Technology and from the Institute of Experimental Biology at Masaryk University in Brno, Czech Republic that conforms to the 2008 Helsinki declaration for human research ethics.

### Participants and the Race

The race participants were notified of the study approximately 2 months beforehand and again 1 week before the race start via e-mail. They were informed about the planned investigation with an indication that their participation was voluntary. No criteria for inclusion/exclusion were used, except that the participants in the study had to finish the race. All volunteers provided a written informed consent and completed an online questionnaire prior to the race. Overall, 632 participants (i.e., 506 men and 124 women) started in the 24-h winter running race. A total of 20 athletes (i.e., 14 men and 6 women) volunteered for the study and finished the race with a full data set.

The 24-h winter running race ‘Adidas 24 h Open Championship of the Czech Republic in winter mountain ultra-marathon of individuals on Lysá Mountain’ started on 25 January 2014 at 10:58 a.m. and finished on 26 January 2014 at 10:58 a.m. in Ostravice, the Czech Republic. One lap measured 11.4 km with 764 m of elevation in a very rugged terrain. The aim of the race was to achieve the highest number of laps, i.e., repeated ascents and descents of the summit of Lysá Mountain within 24 h. The lap count was carried out electronically using chips and only completed laps were taken into account.

The information about weather was obtained from the Czech Hydrometeorological Institute and its professional hydrometeorological station on Lysá Mountain^1^. The average humidity was 88.5%, the precipitation was 0.3 mm and the snow depth was 1 cm. The lowest recorded temperature was -20.6°C and the highest was -7.9°C. The temperature at the top of Lysá Mountain (1323 m) was around -19°C. In addition, gusty wind was blowing, which reduced the air temperature to the windchill values of -28.9°C (25 January 2014) and -18.3°C (26 January 2014).

The refreshments were available at one aid station provided by the organizer, which was located at the start/finish of the circuit. A variety of food and beverages such as hypotonic sports drinks, tea, soup, caffeinated drinks, water, fruit, vegetables, energy bars, bread, soup, sausages, cheese, bread, chocolate and biscuits were available. The runners could also use their own refreshments. Some runners were self-supported; some of them had their support team.

### Measurements and Calculations

The pre-race electronic questionnaire requested information about age and previous running experience in all the participants, as well as male and female runners separately (Table 1). Pre-race testing was carried out on the day of the race start from 7:00 to 11:00 a.m. Post-race measurements were taken immediately after finishing the race. The procedures of the pre- and post-race measurements were identical. The running speed and the number of completed kilometers during the 24 h were obtained from the official on-line results on the race website^2^ (Table 1).

**Table 1:** Anthropometry, pre-race experience, training, average speed and completed kilometers of the finishers.

	All runners (*n* = 20)		Female runners (*n* = 6)		Male runners (*n* = 16)	
	Mean (SD)	Median (IQR)	Mean (SD)	Median (IQR)	Mean (SD)	Median (IQR)
Age (years)	33.2 (11.2)	29.0 (19.0)	37.7 (11.9)	37.5 (20.0)	31.3 (10.3)	26.0 (19.5)
Height (cm)	177.5 (10.5)	174.5 (20.3)	182.6 (8.0)	166.5 (7.8)	182.6 (8.0)	183.0 (15.3)
Years as an active runner (years)	8.0 (7.4)	5.0 (6.3)	8.0 (7.6)	5.0 (7.5)	9.4 (7.8)	5.0 (9.0)
Number of finished ultra-marathons (n)	4.7 (3.2)	3.5 (3.8)	5.5 (3.9)	4.5 (3.0)	5.9 (3.1)	5.0 (4.3)
Mean weekly total training volume (h)^∗^	8.2 (4.5)	7.5 (4.8)	9.0 (5.4)	7.0 (8.5)	6.7 (4.1)	7.5 (4.3)
Mean weekly training volume in running (h)^∗^	6.5 (2.8)	6.0 (3.8)	7.7 (3.9)	6.6 (4.8)	5.6 (2.0)	5.5 (3.3)
Amount of training kilometers in the previous year (km)	1143.4 (569.1)	1.333 (415.0)	1142.9 (652.8)	1350.0 (575.0)	1062.0 (628.5)	950.0 (1003.0)
Longest run in the week preceding the race (km)	33.9 (18.6)	28.0 (10.5)	37.2 (14.2)	29.0 (29.3)	32.5 (20.6)	28.0 (10.5)
Average racing speed (km/h)	4.3 (1.3)	4.3 (2.0)	3.3 (1.1)	3.1 (1.1)	4.7 (1.2)	4.7 (1.4)
Distance completed during 24 h (km)	78.7 (26.8)	70.4 (42.8)	68.5 (24.4)	59.0 (25.7)	83.0 (27.4)	84.7 (48.5)
Completed elevated meters during 24 h (m)	5238.0 (1794.6)	4684.2 (2865.2)	4557.5 (1633.5)	3920.0 (1719.0)	5530.4 (1837.1)	5639.2 (3247.0)

*Results are presented as mean (SD) and median (IQR). ^∗^“Total training volume” means an average sum of training hours if any sport discipline 3 months before the race include running. ^∗^“Training volume in running” means an average sum of training running hours. Participants were instructed to keep a training diary until the start of the race. The training 3 months before the race was recorded*.

Blood samples were drawn from an antecubital vein. The Monovette (plasma gel, 7.5 mL) for chemical analysis and one Sarstedt S-Monovette (EDTA, 2.7 mL) for hematological analysis were kept at 15–25°C and both were sent to the laboratory and analyzed within 6 h. Hematocrit (Hct) was determined using the Sysmex XE 2100 hematology analyzer (Sysmex Corporation, Japan). Relative changes in plasma volume (PV) were calculated from the pre- and post-race Hct values according to the equation of van Beaumont (Van Beaumont, 1972). Plasma creatinine (Pcr), CK and PU were determined using the Modular SWA biochemical analyzer, Module P + ISE (Hitachi High Technologies Corporation, Japan, Roche Diagnostic). Creatinine clearance (CrCl) was calculated using the Cockcroft and Gault formula (Cockcroft and Gault, 1976). For the evaluation of blood parameters, laboratory reference values for adults (Kratz et al., 2004) were used. However, strenuous exercise may have a profound effect on laboratory parameters (Kratz et al., 2002). Therefore, we also used the modified reference ranges for basic biochemical and hematological laboratory parameters originally designed for marathon runners (Kratz et al., 2002) (pre-race PU 5–27 mg/dL, post-race PU 11–28 mg/dL; pre-race Pcr 0.7–1.3 mg/dL, post-race Pcr 0.7–1.9 mg/dL; pre-race CK 19–245 U/L, post-race CK 0–2377 U/L, all values are identical for males and females).

One trained investigator undertook all the body composition assessments. Every participant underwent anthropometric measurements in order to determine total BM, height, skinfold thicknesses and limb circumferences to calculate SM, fat-free mass (FFM), BF and percentage BF (%BF). Height was determined using a stadiometer (TANITA HR 001, Tanita Europe B.V., Amsterdam, Netherlands) to the nearest 1 cm. Total BM was measured using a calibrated commercial scale (Tanita BC-351, Tanita Corporation of America, Inc.) placed on a hard, level surface to the nearest 0.1 kg. Each athlete was required to have approximately the same clothes for both the pre- and post-race measurements, all runners were barefoot and they had to empty their urinary bladder prior to the anthropometric measurements. After completing the race, before eating or drinking, all subjects were weighed again on the same scale. Body composition assessments were undertaken using ISAK (International Society for the Advancement of Kinanthropometry) protocols (Marfell-Jones et al., 2006). The circumferences of mid-upper arm, mid-thigh and mid-calf were measured on the right side of the body to the nearest 0.01 cm using a non-elastic tape measure (KaWe CE, Kirchner und Wilhelm, Germany). The circumference of the upper arm was measured in the middle of the upper arm (between the acromion and olecranon) in an anatomical position to the nearest 0.1 cm, the circumference of the thigh was taken at the level where the skinfold thickness of the thigh was measured (20 cm above the upper margin of the patella), and the circumference of the calf was taken at the level of the largest circumference of the calf. Skinfold thicknesses were measured for eight skinfolds: pectoralis, midaxillary (vertical), triceps, subscapular, abdominal (vertical), suprailiac (in the anterior axillary line), thigh and calf using a skinfold caliper (Harpenden skinfold caliper, Baty International Ltd) to the nearest 0.2 mm at the right side of the body according to Lee et al. (2000). The skinfold measurements were taken for all the eight skinfolds three times by the same investigator. The mean value of the three measurements was then used for the analyses. The timing of the skinfold measurements was standardized to ensure reliability. In accordance with Becque et al. (1986), readings were performed 4 s after applying the caliper. An inter-tester reliability check was conducted on 27 male and 11 female runners (Knechtle et al., 2010a). No significant differences were observed between the two trials for the sum of eight skinfolds (*p* > 0.05). The intra-class correlation within the two judges for both men and women for all anatomic sites was high (ICC > 0.9). Moreover, we have used the ISAK landmarking of points for the measurement (Marfell-Jones et al., 2006). Body composition related variables such as BF, FFM and SM were estimated using reliable and valid equations. The SM was calculated using the following formula: SM = Ht x (0.00744x CAG^2^ + 0.00088 x CTG^2^ + 0.00041 x CCG^2^) + 2.4 x sex – 0.048 x age + race + 7.8, where Ht = height, CAG = skinfold-corrected upper arm girth, CTG = skinfold-corrected thigh girth, CCG = skinfold corrected calf girth, sex = 1 for male and 0 for female runners, race = 0 for white, according to Lee et al. (2000). The FFM in male participants was calculated using the anthropometric method of Stewart and Hannan (2000) in which BM, the abdominal, suprailiac and thigh skinfold measurements of the participants are used. Stewart and Hannan (2000) calculated the coefficient of determination for this equation to be 0.96 and found a standard error of the estimate of 1.74 kg for the last-mentioned equation. The BF was determined by subtracting FFM from BM. Female BF was calculated using an equation for female athletes (Warner et al., 2004), in which the participants’ BM, abdominal and thigh skinfold values are used. A valid correlation coefficient of 0.98 was reported by Warner et al. (2004), for the directly measured and indirectly calculated FFM values of the participants. Male %BF was calculated using the following anthropometric formula: %BF = 0.465 + 0.180(Σ7SF) – 0.0002406(Σ7SF)^2^ + 0.0661(age), where Σ7SF = sum of the mean skinfold thicknesses of pectoralis, midaxillary, triceps, subscapular, abdomen, suprailiac and thigh, according to Ball et al. (2004a). Female %BF was estimated using a specific equation for women (Ball et al., 2004b): %BF = -6.40665 + 0.41946 (Σ3SF) – 0.00126 (Σ3SF)^2^ + 0.12515 (Hip) + 0.06473, where Σ3SF was taken as the sum of the three skinfold thicknesses of the triceps, suprailiac, and front thigh skinfolds; and Hip was the circumference of the hip. TBW was measured using a multiple-frequency bioelectrical impedance analyzer (InBody 720, Biospace, Seoul, South Korea) in accordance with Bedogni et al. (2002) and Chapman et al. (1992). Inbody 720 has a tetra polar 8-point tactile electrode system performing 30 impedance measurements at each session by using six different frequencies (1, 5, 50, 250, 500, and 1,000 kHz) at each of the five segments (right arm, left arm, trunk, right leg, and left leg).

During pre-race sample collection, the participants were informed about the volumes of the cups offered at the aid station. The runners were instructed to record their fluid intake (including fluids the runners carried on them and consumed during the race) and beverage quantities during the race on recording sheets on their own or with the help of their support team and they were obliged to recall their fluid intake at the finish during post-race measurements. Moreover, the fluid intake during the race was continuously recorded by assistants at the aid station who marked the number of cups consumed by the runners. Following each loop, the runners were also asked to recall the fluids they drank. Final fluid intake was estimated based on the runners’ reports and additional information provided by the assistants at the aid station. At the end of the race, during post-race measurements, the runners were asked again by a trained nutritionist to recall their whole fluid intake during the race. Information from the support crew and/or the runner allowed for discrepancies in their reports, which may have occurred due to tiredness and were then discussed in order to increase the accuracy of the results. Fluids that were part of a meal or snack were not recorded. The event website did not provide the athletes with any special advice on what and how much they should drink during the race. Hydration status was classified according to the criteria established by Noakes et al. (2005) with overhydration classified as any weight gain above the initial BM, euhydration as stable BM or a decrease in BM of 0.01–3.0%, and dehydration as any decrease in BM greater than 3.0%.

### Statistical Analysis

Descriptive statistics (mean, standard deviation plus median, interquartile range, as the data was often not normally distributed) were calculated for pre- and post-race values, absolute and percentage changes of BM, BF, SM, TBW, PU, Pcr, CrCl, PV, and CK parameters. Normal distribution was verified by Anderson-Darling’s test for normality. The data was often not normally distributed, and even if it passed the normality test, there was a high risk of type II error due to the small size of the groups. Comparisons between the male and female groups were made using unpaired non-parametric two-sample Wilcoxon test (also known as Wilcoxon rank sum test or Mann-Whitney test). Pair data, especially information on the pre-and post-race values within each group, was analyzed using paired one sample Wilcoxon rank sum test. The dependencies between the individual characteristics were tested using the Spearman’s correlation coefficient with 95% confidence interval by Bonett and Wright (2000). The data was processed using the MINITAB 17 statistical software. Statistical significance was set at *p* < 0.05 for all analyses.

## Results

### Body Mass, Body Fat and Skeletal Muscle Mass

**FIGURE 1:**
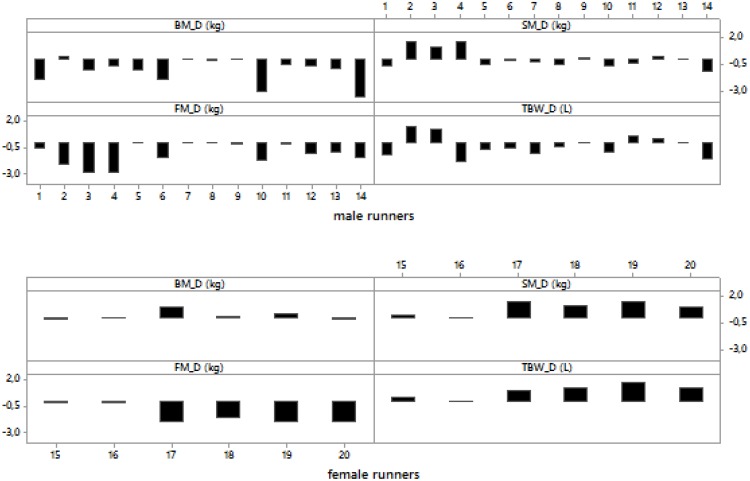
Post- minus pre-race differences in body mass, skeletal muscle mass, body fat and total body water in male and female runners.

**FIGURE 2:**
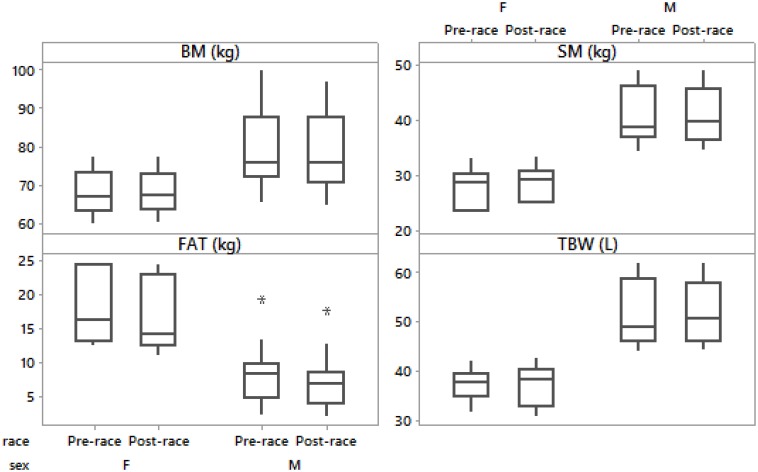
Box-plot of post- minus pre-race differences in body mass, skeletal muscle mass, body fat and total body water in male and female runners.

Pre- and post-race BM were significantly higher in the male than in the female group (*p* = 0.02, *p* = 0.03, respectively). The average BM decreased significantly post-race (Table 2) with the individual BM losses ranging from -0.1 to -4.9% (mean). BM decreased in men (Table 3), but remained stable in women (Table 4) (Figure 1, 2). Body composition parametres expressed by median and interquartile range are shown in Table 2 for the whole group (*n* = 20) an in Tables 3, 4 for female and male runners separately.

**Table 2:** Body composition, blood parameters (*n* = 20).

	Pre-race		Post-race		Absolute change		Percentage change		*P*-value
	Mean (SD)	Median	Mean (SD)	Median	Mean (SD)	Median	Mean (SD)	Median	
		(IQR)		(IQR)		(IQR)		(IQR)	
Body mass (kg)	76.0 (10.3)	73.7 (13.4)	75.2 (9.9)	72.4 (13.8)	-0.8 (1.1)	-0.5 (1.0)	-1.0 (1.4)	-0.6 (1.3)	0.002*
Body fat (kg)	11.2 (6.5)	9.7 (8.3)	10.0 (6.3)	8.5 (9.4)	-1.2 (0.9)	-1.2 (1.7)	-11.7 (10.8)	-10.2 (11.0)	<0.001*
Body fat (%)	15.1 (9.3)	13.0 (11.1)	13.6 (8.8)	12.0 (11.9)	-1.5 (1.3)	-1.3 (2.6)	-10.9 (10.8)	-10.2 (10.6)	<0.001*
Skeletal muscle mass (kg)	36.9 (7.7)	37.3 (13.9)	37.1 (7.3)	37.1 (12.9)	0.3 (0.9)	0.0 (1.6)	1.0 (2.8)	-0.0 (4.9)	0.401
Total body water (L)	47.1 (8.6)	47.8 (16.0)	47.4 (8.9)	46.9 (15.4)	0.3 (3.1)	-0.1 (2.0)	0.7 (7.2)	-0.1 (3.7)	0.845
Plasma urea (mmol/L)	4.9 (1.5)	4.6 (1.6)	8.5 (2.7)	8.6 (3.5)	3.6 (2.0)	3.3 (2.6)	76.2 (44.6)	73.0 (61.4)	<0.001*
Plasma creatinine (μmol/L)	74.3 (13.1)	72.5 (13.5)	79.4 (15.4)	81.5 (24.0)	5.2 (6.2)	4.0 (12.2)	6.9 (8.7)	6.4 (15.1)	0.004*
Creatine kinase (U/L)	150.0	140.9	5545.2	1976.0	5395.2	1759.0	3380.3	1496.0	<0.001*


	(61.8)	(68.2)	(9318.4)	(5066.0)	(9306.7)	(5110.0)	(5601.5)	(2495.0)	
Creatinine clearance	131.6 (25.8)	128.9 (23.5)	123.5 (29.3)	123.9 (27.6)	-8.1 (9.7)	-8.9 (14.3)	-6.7 (7.9)	-6.2 (14.9)	0.002*

*Results are presented as mean (SD) and median (IQR), ^∗^*p* < 0.05*.

**Table 3:** Body composition, blood parameters in female runners (*n* = 6).

	Pre-race		Post-race		Absolute change		Percentage change		*P*-value
	Mean (SD)	Median	Mean (SD)	Median	Mean (SD)	Median	Mean (SD)	Median	
		(IQR)		(IQR)		(IQR)		(IQR)	
Body mass (kg)	68.0 (67.4)	67.4 (9.8)	67.9 (5.8)	67.2 (9.4)	-0.1 (0.3)	-0.2 (0.4)	-0.1 (0.4)	-0.2 (0.6)	0.600
Body fat (kg)	17.9 (5.4)	16.2 (11.4)	16.6 (5.5)	14.3 (10.4)	-1.3 (0.9)	-1.8 (1.7)	-7.8 (5.4)	-9.9 (10.8)	0.036*
Body fat (%)	26.4 (7.2)	25.7 (13.9)	24.3 (6.9)	22.7 (14.2)	-2.1 (1.5)	-2.6 (3.2)	-8.0 (5.6)	-10.6 (11.3)	0.036*
Skeletal muscle mass (kg)	27.7 (3.7)	28.7 (6.7)	28.6 (3.2)	29.3 (5.8)	0.9 (0.7)	1.1 (1.4)	3.4 (2.8)	3.7 (6.0)	0.059
Total body water (L)	37.2 (3.5)	37.7 (7.5)	37.1 (4.3)	38.3 (7.6)	-0.1 (2.6)	0.8 (1.7)	-0.3 (7.2)	2.1 (4.8)	0.208
Plasma urea (mmol/L)	4.6 (1.1)	4.5 (1.9)	8.1 (2.3)	7.2 (3.2)	3.4 (2.0)	3.1 (2.3)	77.4 (40.1)	85.0 (65.6)	0.036*
Plasma creatinine (μmol/L)	64.7 (9.6)	63.5 (17.8)	69.0 (15.6)	63.5 (30.3)	4.3 (8.3)	1.5 (16.8)	6.0 (11.6)	2.7 (22.9)	0.463
Creatine kinase (U/L)	118.9	122.1	2009.0	1256.0	1890.0	1103.0	1643.0	781.0	0.036*


	(36.6)	(58.8)	(2248.0)	(2192.0)	(2248.0)	(2154.0)	(2101.0)	(1754.0)	
Creatinine clearance	114.4 (27.4)	113.3 (41.4)	110.4 (32.0)	118.0 (59.5)	-4.0 (10.2)	-2.7 (17.7)	-4.5 (10.1)	-1.4 (19.7)	0.402

*Results are presented as mean (SD) and median (IQR), ^∗^*p* < 0.05*.

**Table 4:** Body composition, blood parameters in male runners (*n* = 14).

	Pre-race		Post-race		Absolute change		Percentage change		*P*-value
	Mean (SD)	Median	Mean (SD)	Median	Mean (SD)	Median	Mean (SD)	Median	
		(IQR)		(IQR)		(IQR)		(IQR)	
Body mass (kg)	79.5 (9.9)	75.8 (15.6)	78.4 (9.7)	75.8 (16.8)	-1.1 (1.1)	-0.8 (1.8)	-1.4 (1.4)	-1.1 (2.0)	0.003*
Body fat (kg)	8.2 (4.5)	8.3 (5.1)	7.2 (4.3)	6.9 (4.7)	-1.1 (1.0)	-1.0 (1.6)	-13.4 (12.2)	-10.6 (15.9)	0.001*
Body fat (%)	10.2 (4.6)	10.6 (6.6)	9.0 (4.5)	9.3 (7.4)	-1.2 (1.2)	-1.1 (1.7)	-12.2 (12.4)	-9.0 (16.9)	0.003*
Skeletal muscle mass (kg)	40.8 (5.0)	38.8 (9.4)	40.8 (5.0)	39.8 (9.1)	-0.0 (0.9)	-0.3 (1.1)	-0.0 (2.2)	-0.7 (2.5)	0.572
Total body water (L)	51.3 (6.2)	50.2 (11.7)	51.8 (6.3)	50.6 (11.7)	0.5 (3.3)	-0.5 (1.4)	1.1 (7.4)	-1.0 (2.8)	0.224
Plasma urea (mmol/L)	5.1 (1.6)	4.7 (1.6)	8.8 (2.8)	8.8 (4.3)	3.7 (2.0)	4.0 (2.9)	75.7 (47.8)	64.3 (64.2)	0.001*
Plasma creatinine (μmol/L)	78.4 (12.5)	77.0 (20.5)	83.9 (13.5)	83.0 (19.8)	5.5 (5.4)	4.5 (8.3)	7.2 (7.7)	6.4 (8.1)	0.006*
Creatine kinase (U/L)	163.3	149.7	7061.0	2521.0	6897.0	2376.0	4124.0	1978.0	0.001*


	(66.6)	(85.3)	(10803.0)	(5637.0)	(10794.0)	(5542.0)	(6493.0)	(2649.0)	
Creatinine clearance	139.0 (22.0)	131.8 (25.7)	129.1 (27.3)	124.6 (33.7)	-9.9 (9.2)	-10.5 (10.3)	-7.6 (6.9)	-6.9 (8.9)	0.003*

*Results are presented as mean (SD) and median (IQR), ^∗^*p* < 0.05*.

Fourteen (70%) runners were euhydrated (50% women and 79% men), two (10%) runners (14% men and 0% women) were dehydrated (-3.1 and -4.9%) and four (20%) runners (7% men and 50% women) were overhydrated (from 0.2 to 1.5%) post-race according to the classification based on BM changes (Δ) by Noakes et al. (2005).

Female runners drank 300.0 (57.7) mL/h and male runners drank 464.3 (201.3) mL/h without any significant difference between the sexes (*p* > 0.05). Fluid intake was not associated with Δ BM, Δ SM, Δ BF, Δ TBW or Δ PV (*p* > 0.05). Pre- and post-race BF were significantly higher in male than female group (*p* = 0.003, *p* = 0.002, respectively). BF decreased significantly post-race (Table 2) in both men (Table 3) and women (Table 4) without a significantly higher BF decrease in male compared to female ultra-marathoners (*p* > 0.05) (Figure 1, 2).

Pre- and post-race SM were significantly higher in males than in females (*p* = 0.001, *p* = 0.001, respectively). SM remained stable in all competitors (Table 2), both men and women (Tables 3, 4) (36% of male runners with a non-significant increase and 64% with a non-significant decrease in SM; 83% of female runners with a non-significant increase and 17% with a non-significant decrease) (Figure 1, 2) without any significant differences between the female and male ultra-marathoners; however, on the border of statistical significance (*p* = 0.053) (Figure 1, 2). Δ BM was related to Δ SM (*r* = 0.58, *p* = 0.007, confidence interval (CI) = [0.15; 0.83]). Δ BF was negatively associated with Δ SM (*r* = -0.65, *p* = 0.002, CI = [0.25; 0.86]). Δ BM was not related to Δ BF (*p* > 0.05).

### Plasma Volume and Total Body Water

**FIGURE 3:**
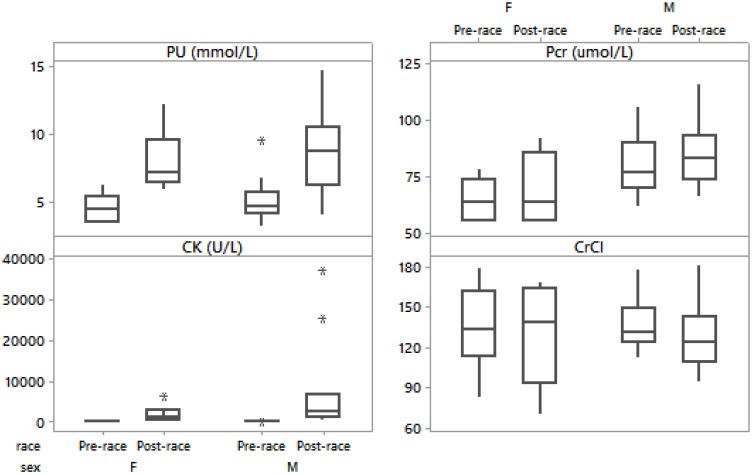
Box-plot of post- minus pre-race differences in plasma urea, plasma creatinine, creatine kinase, creatinine clearance in male and female runners.

PV increased by 17.6 (10.2)% (mean, standard deviation) and [(15.6 (14.5) (median, interquartile range - IQR)] in all runners, 18.3 (12.3)% (mean, standard deviation) and [(15.4 (19.2) (median, IQR)] in male and 17.3 (3.8)% (mean, standard deviation) and [(16.5 (6.3) (median, IQR)] in female runners without any significant difference between the sexes (*p* > 0.05). Pre- and post-race TBW were significantly higher in males than in females (*p* = 0.001, *p* = 0.001, respectively). TBW remained stable in all runners (Table 2), both female (Table 4) (non-significant increase in 67% and decrease in 33% of the female runners) and male (Table 3) (non-significant decrease in 57%, increase in 29% and stable TBW in 14%), without a significant difference between the sexes (*p* > 0.05) (Figure 3). Δ BM was related to Δ TBW (*r* = 0.59, *p* = 0.007, CI = [0.16; 0.83]). Δ SM was associated with Δ TBW (*r* = 0.51, *p* = 0.021, CI = [0.06; 0.79]). Δ TBW was not related to PV or Δ BF (*p* > 0.05).

### Plasma Urea, Plasma Creatinine, Creatinine Clearance and Creatine-Kinase

There were no significant differences in pre- and post-race PU in the male compared to the female group (*p* > 0.05). The overall PU significantly increased in all runners (Table 2). PU increased in both men (Table 3) and women (Table 4 and Figure 3). Post-race PU values were higher than the reference ranges (Kratz et al., 2002) in five male runners (24%) (10.2–14.7 mmol/L).

Pre- and post-race Pcr were significantly higher only pre-race in the male than in the female group (*p* = 0.04, *p* > 0.05), respectively). The overall Pcr significantly increased (Table 3). Pcr significantly increased in men (Table 3) and non-significantly in women (Table 4 and Figure 3); however, without significant difference between sexes (*p* > 0.05). Post-race Pcr levels were not higher than the reference ranges for marathoners reported by Kratz et al. (2002) and three women (50%) showed lower concentrations pre- (55.0–58.0 μmol/L) and post-race (55.0–59.0 μmol/L).

There were no significant differences in pre- and post-race CK in the male compared to the female group (*p* > 0.05). The overall CK increased significantly (Table 3) without significant difference between sexes (*p* > 0.05). Ten (50%) runners had CK levels over 2,000 U/L (nine males and one female, thereof two (10%) male runners had CK levels above 10,000 U/L). CK increased significantly in both men (Table 3) and women (Table 4). Post-race CK was higher (Kratz et al., 2002) in seven (50%) male (2,810–37,265 U/L) and one (17%) female runner (6,502 U/L). There were no significant differences in pre- and post-race CrCl in the male compared to the female group (*p* > 0.05). CrCl decreased (Table 2), in both men (Table 3) and women (Table 4) without significant difference between sexes (*p* > 0.05). The decrease in BM was negatively related to the increase in CK (*r* = -0.71, *p* < 0.001, CI = [0.34; 0.89]). Δ CK was associated with the number of completed kilometers during the 24 h (*r* = 0.77, *p* < 0.001, CI = [0.45; 0.92]). Post-race PU was related to the number of completed kilometers during the 24 h (*r* = 0.56, *p* = 0.009). Δ Pcr was positively related to Δ PU (*r* = 0.64, *p* = 0.002, CI = [0.23; 0.86]). The decrease in CrCl was negatively associated with the increase in PU (*r* = -0.72, *p* < 0.001, CI = [0.36; 0.89]) and the increase in CK (*r* = -0.48, *p* = 0.032, CI = [0.02; 0.77]).

## Discussion

The aim of the study was to quantify body composition characteristics and parameters associated with skeletal muscle damage and renal function in 24-h ultra-marathoners competing under extremely cold winter conditions. Both BM and BF decreased, whereas SM and TBW remained stable in male runners. In female runners, BF decreased, whereas BM, SM and TBW remained stable. Biomarkers of skeletal muscle damage, exertional rhabdomyolysis and transient renal injury increased post-race.

### Body Mass and Body Fat Changes

Body fat decreased significantly in all participants, as well as when we investigate male and female runners separately. On the contrary, BM significantly decreased only in male runners. The 24-h ultra-marathoners competing in hot summer weather (10–31°C) presented in a study by Knechtle et al. (2011b) showed a higher BM loss (-3.1%) in 13 out of 15 runners. Kao et al. (2008) recorded BM losses ranging from -0.8 to -11% in seven runners (five males and two females) during a 24-h ultra-marathon held on an oval track with temperatures ranging from 12 to 15°C, too. BM decreased in the range from 0 to -6.2% in a study by Fellmann et al. (1988) in eight (seven male and one female) 24-h ultra-marathoners during a race at temperatures of 4 ± 1°C. By contrast, BM losses were similar (-1.6%) in twenty-five (19 male and 6 female) 24-h ultra-endurance runners with ambient temperature ranging from 0°C to 20°C (Costa et al., 2014). The decrease in BM was significant only in male in contrast to female runners in a study on 24-h ultra-runners competing in a summer race at temperatures between 10 and 18°C, but with heavy rain (Chlíbková et al., 2015) and the results were comparable with the BM losses recorded in the present study. Other investigations during ultra-endurance races have also reported stable BM (Knechtle et al., 2008) as in present female runners or even increased BM (Raschka and Plath, 1992) despite changes in body composition and fluid retention was likely masking greater BM and body composition changes. Females have tendency for attenuated BM and BF losses during endurance races (Weitkunat et al., 2012; Baur et al., 2016). According to Hoffman et al. (2018), the change in BM does not exactly reflect the changes in hydration status and BM change is only one of several variables responsible for hydration status changes. Metabolic response to cold is higher in men then in women, and men usually have lower pre-race subcutaneous fat than women (Stevens et al., 1987), similarly as in the current study. Male runners also showed higher pre-race muscle mass than female runners, so we could expect differences between men and women in metabolism. Despite these facts, female BF losses were 1.3 kg, even more than 1.02 kg in female racers in the study of athletes under arctic conditions (Chapman et al., 1992). Moreover, there were no significant differences in BF loss between the male and female runners.

### Maintenance of Body Fluid Homeostasis

Total body water and SM remained stable in both sexes. No changes in fat free mass and TBW were noted during The Yukon Arctic Ultra in the study by Schalt et al. (2018). A study during an ultra-distance race in cold weather in Alaska (17 male and 3 female athletes) with temperatures ranging from -14 to -2°C (Stuempfle et al., 2003) described a mean BM loss of 1.2 kg; similarly as in the present male runners. The subjects were hyponatremic with an absolute BM change of 0.8 kg (normonatremic group with 1.9 kg) in another study from the same winter race in Alaska (14 male and 2 female athletes) (Stuempfle et al., 2002). Exercise in colder environment leads to the occurence of cold-induced diuresis resulting in additional losses in body water and BM (Weitkunat et al., 2012). Therefore, Stuempfle et al. (2003) considered a lower BM loss in their study as an evidence of fluid overload. However, TBW was probably maintained due to the production of metabolic water during fuel oxidation and due to the release of water with the breakdown of muscle and liver glycogen (Maughan et al., 2007; Hoffman and Stuempfle, 2014) in the current study. According to BM changes by Noakes et al. (2005), most of the present male runners were euhydrated. In addition, the reported fluid intake was not associated with changes in BM, SM, BF or PV. The average TBW remained stable and BM decreased, thus we did not presume overhydration in the present male 24-h ultra-marathoners. Concerning female runners, one half of them was classified as overhydrated post-race according to the classification based on BM changes (Noakes et al., 2005). Notwithstanding; their changes in PV, BM, skeletal muscle or TBW were not associated with fluid intake. A BM loss of -1% could be expected from fat use (Stuempfle et al., 2011) and pre-race BM is typically ∼1% higher immediately before the start of the race in comparison with the day before (Hoffman et al., 2013). If we define BM change ≥1% as overhydration (Hoffman and Stuempfle, 2014), only one female runner can be considered overhydrated post-race. Moreover, no significant differences between the male and female group regarding fluid intake were found. In the present 24-h ultra-runners no fluid overload occurred (Chlíbková et al., 2019) and therefore no disturbance of the body fluid homeostasis or of any other dimension could be determined. Pre-race carbohydrate loading, non-standardized diet, substrate intake, utilization and metabolism during the race and the extreme nature of this event probably altered the relationship between BM and body water (Olsson and Saltin, 1970). Moreover, we presume that body composition reductions were most likely due to substrate losses (Maughan et al., 2007) and were not indicative of the significant body water losses in the present study. These findings are consistent with a 48-h period of energy restriction without fluid restriction and in response to cold exposure, when BM loss occurred without changes in hydration status (Costa et al., 2010).

### Total Body Water Associated With Body Mass and Skeletal Muscle Mass Changes

The next important finding was that the changes in BM were not related to the changes in BF; however, they were significantly associated with SM and TBW changes. The same findings were noted by Baur et al. (2016) in a study of participants in a 3-day multistage ultra-endurance triathlon (with temperatures ranging from 1.1°C to 26.7°C). The authors reported very close associations between BM and TBW changes and fat-free mass changes. TBW remained stable in both sexes; nevertheless, there was a non-significant increase in 25% of male and 67% of female runners in the current study. The reasons may be preservation of TBW and attenuated sweat response in females and thereby reduced fluid fluctuation (Inoue et al., 2014). An ultra-endurance performance often leads to an increase in TBW (Fellmann et al., 1988; Knechtle et al., 2009). However, apart from fluid overload, various mechanisms could lead to TBW retention (Lehmann et al., 1995; Mischler et al., 2003; Maughan et al., 2007). The production of metabolic water is linked to the oxidation of fatty acids (Milledge et al., 1982) and carbohydrates and water from glycogen degradation in muscles and liver (Pastene et al., 1996). The body probably tries to maintain circulating volume and metabolic processes so that energy supply could be preserved for prolonged exercise. Due to the association of TBW, SM and BM in the present runners, the increase in TBW seems to be distributed in the SM. Intravascular water is increased by water of oxidation, gastrointestinal water absorption, water released from glycogen and also by peripheral edemas (Hoffman et al., 2018). The activation of the renin-angiotensin system and plasma aldosterone were described by Milledge et al. (1982) as a reason for the increased PV and body water retention leading to peripheral edemas. Knechtle et al. (2009) presumed that peripheral edemas developed in the skeletal muscle. Water associated with peripheral edemas could contribute to the increase in TBW, so an equal amount of water had to be gained to maintain hydration (Hoffman et al., 2018). Taking into account significant losses in BF with no relationship to changes in BM in the present study and lower BM losses in male and stable BM in female runners despite the heavy load, the total degradation of glycogen stores and extremely cold weather conditions, real BM and composition changes could be masked by alternations in fluid dynamics and the probable occurrence of subsequent peripheral edemas (Costa et al., 2014; Baur et al., 2016). Nevertheless, although PV significantly increased, potential edema formation cannot be confirmed as foot volume was not measured. Calculations by Hoffman et al. (2018) showed that a runner with even lower average BM that the present runners must lose at least 1.9% of BM to maintain body water balance. Endogenous substrate mass probably decreased more, and the missing kilograms could be explained by the shifts in the TBW presumably by the formation of edemas in runners with increased SM. Swelling and inflammatory responses probably caused an increase in internal fluid pressure (Nosaka and Clarkson, 1996) and led to muscle thickness increase accompanied by edemas. The reasons and mechanisms for the formation of peripheral edemas are not fully understood and a very likely explanation for the development of peripheral edema is fluid overload (Bracher et al., 2012; Cejka et al., 2012), however, this was not confirmed in the present study. Nevertheless, peripheral edemas can be caused by the movement of water from the intracellular to the extracellular compartments rather than by the increase of TBW (Milledge et al., 1982; Lehmann et al., 1995). The swelling might also be a high protein interstitial space fluid swelling (Vitiello et al., 2015) and may be associated with markers of skeletal muscle damage (Knechtle et al., 2009).

### Body Fat Decrease Associated With Skeletal Muscle Mass Increase

An interesting fact was the finding that runners with a higher decrease in percentage BF showed a higher increase in SM. Investigations in athletes exercising in extremely cold conditions for extended periods (Coker et al., 2017; Johannsen et al., 2018; Schalt et al., 2018; Shepard, 1985, 1993) found significant reductions in BF, but no change in fat free mass, despite sustained negative energy balance, similarly as in the present study. Moderate negative associations between fat-free mass and percentage BF have been reported by Baur et al. (2016). Shepard (1985, 1993) noted that metabolic adaptations to exercise in the cold were associated with increased BF metabolism, which is stimulated by the energy cost of lean tissue synthesis. A possible factor contributing to BF decline and non-significant BM loss in present female runners could also be the energy cost of synthesizing new lean tissue (Shepard, 1985). Additional studies performed with the athletes in the Alaska Mountain Wilderness Ski Classic under similar arctic winter conditions also showed preservation and even increase in the lean tissue mass despite high energy expenditure, as measured by dual-energy X-ray absorptiometry (Johannsen et al., 2018). This pattern of response occurs in subjects with higher initial BF or physically weaker (Shepard, 1985). Coker et al. (2017) reported almost complete muscle preservation under extreme conditions of prolonged cold exposure during the Yukon Arctic Ultra in athletes at all distances, also at the shortest distance with twice less kilometers than the distance covered on average by the present runners during 24 h. Higher BF and BM loss could then be masked by the synthesis of a similar mass of lean tissue in some of the present 24-h runners, both males and females. Results suggest that runners may utilize a combination of training, pacing strategies and nutrition that enable adaptive responses to the environmental stress (Schalt et al., 2018). Future studies should be directed at measuring dietary intake with the molecular components of protein metabolism and/or training methods that may be responsible for the lean BM preservation despite sustained high levels of caloric expenditure and extremely cold conditions.

### Parameters Detected Skeletal Muscle Mass Damage and Transient Renal Impairment

Plasma urea, Pcr and CK significantly increased post-race. CK increased as an indicator of damage to the organs and exertional rhabdomyolysis as the most common cause of acute renal injury and acute renal failure in runners (Patel et al., 2009). CK levels over approximately 2,000 U/L used as the criterion for statin myopathy (Thompson et al., 2003) were reported in 50% of the present runners and CK above 10,000 U/L, diagnostic of rhabdomyolysis (Sinert et al., 1994), were shown by 10% of them. These results are in accordance with other studies investigating marathoners and ultra-marathoners. Kratz et al. (2002) reported that Pcr was outside the standard reference ranges in 30% and CK in 81% of the marathoners. Serum activity of CK increased 70-fold in a study of 24-h ultra-marathoners by Waśkiewicz et al. (2012) due to mechanical damage or increased membrane permeability. Post-race PU values were higher than the reference ranges in 24% of the male runners. An increase in PV and PU could be explained by protein catabolism with the occurence of hypoproteinemic edemas (Lehmann et al., 1995) due to proteolysis during prolonged exercise (Mischler et al., 2003). Moreover, the runners with a higher number of completed km during the 24 h showed a higher increase in PU and CK post-race. Although SM remained stable, we found a non-significant decrease in nearly two thirds of resent male runners. This variability in SM changes may have caused the overall non-significant difference between the post- and pre-race values and also zero association between decrease in BF and decrease in BM. SM seems to decrease in ultra-endurance races without breaks (Knechtle and Bircher, 2005). Eccentric running led to a damage of SM and consequently to its loss due to the depletion of intramyocellular lipid and/or muscle triglyceride stores caused by a substantial energy deficit (Knechtle and Bircher, 2005). The loss in lean BM including water, proteins, glycogen and non-bone minerals was explained by a decrease in muscle density caused by glycogen loss in Ironman triathletes (Mueller et al., 2013). The reduction in glycogen stores together with water bound to glycogen could result in the loss of SM as well as in BM loss in the present runners.

Plasma creatinine changes were associated with changes in PU in the present runners and probable development of swelling could be also due to a decline in renal function caused by SM damage (Uberoi et al., 1991). Biochemical criteria for acute renal injury require Pcr concentration higher than 2.0 mg/dL and 1.5 times the estimated baseline (Clarkson et al., 2006), and acute renal failure is defined by Pcr >3-fold from baseline or >4 mg/dL with an acute rise of 0.5 mg/dL or greater. None of the runners reached these values. Post-race Pcr levels were within the reference ranges (Kratz et al., 2002) and the moderate decline of renal functions seemed to be a physiological response to ultra-marathon running. Moreover, acute renal failure is multifactorial and caused by the combined effect of rhabdomyolysis, dehydration, hypotension, hyperuricemia, etc. (Uberoi et al., 1991). However, we found a negative association between the decrease in creatine clearance and the increases in both PU and CK. The increased Pcr production was probably caused by catabolic metabolites of skeletal muscle damage and we presume transient impairment of the kidney function.

### Limitations

This study has a few limitations worth noting. We took the skinfold measurement three times by the same investigator; however, limb circumferences were measured only once, and this might have influenced our estimation of SM. By contrast, in agreement with the development of anthropometric equations and measurement protocols for specific populations, such as athletes, in recent decades, it seems that the anthropometry technique could be more reliable in field measurements than BIA (Knechtle et al., 2011c). Another limitation of our study was the fact that it was not possible to determine body water changes through the isotope dilution method (Nolte et al., 2011) and we used BIA measurement similarly as Chapman et al. (1992) in his field testing of athletes competing in Alaska under arctic conditions; as Coker et al. (2017) investigated metabolic responses to the Yukon Arctic Ultra and Schalt et al. (2018), who used BIA for TBW measurement during the Yukon Arctic Ultra. A correct estimation of body water with BIA requires equilibrated fluid shifts and unchanged electrolyte concentrations (Pialoux et al., 2004) and TBW remains steady when BM losses are between 2–3% after long-duration exercise (Nolte et al., 2011; Tam et al., 2011). Thus, with an average BM loss of 1.4% in males and stable BM in the female group, BIA appeared to have produced physiologically relevant values (Hew-Butler et al., 2015). Besides that, we were primarily interested in the changes between pre- and post-race values and not in the absolute values and plasma osmolality remained unchanged in present 24-h ultra-runners (Chlíbková et al., 2019). In addition, we did not measure energy expenditure and energy intake, because this would be problematic due to the character of this field study and extreme weather conditions which caused great physical exhaustion and inability of some competitors to cooperate in this direction. Finally, we did not mention the aerobic fitness (VO2max) of the participants because the racers were from the various places around the Czech Republic and it was impossible to do any pre-race measurements in a laboratory.

## Conclusion

The 24-h winter mountain running race under extremely cold conditions led to a significant decrease in BF in both sexes, whereas SM and TBW remained stable. Nevertheless, biomarkers of skeletal muscle damage, and renal injury increased post-race and were related to the damage of SM with transient impaired renal function. We found significant association between SM and TBW changes, which could mask real body composition changes in both men and women and the formation of edemas has to be taken into consideration. Simultaneously, we found a significant association between fat mass decrease and SM increase, so we must also take into account the possibility of lean tissue synthesis which may have concealed higher BF and BM losses.

## Ethics Statement

The study received ethical approval from the institutional review boards of the Centre of Sport Activities at the Brno University of Technology and from the Institute of Experimental Biology at Masaryk University in Brno, Czech Republic that conforms with the 2008 Helsinki declaration for human research ethics.

## Author Contributions

DC designed the study, collected the data, and wrote the manuscript. AŽ collected the data. TR and BK helped with designing the study and drafting the manuscript. JB performed statistical analyses. All authors have read and approved the final manuscript.

## Conflict of Interest Statement

The authors declare that the research was conducted in the absence of any commercial or financial relationships that could be construed as a potential conflict of interest.

## Footnotes

1 http://www.lysahora.cz/dokumenty/seznam.phtml?od=50\&limit=25\&id_objektu=107685\&slozka=7

2 www.lh24.cz/history/2014/lh24/?language=en-GB
